# The mediating role of moral disengagement in the relationship between dark personality trait and perceptions for female performance athletes: the role of age, gender, and physical activity level

**DOI:** 10.3389/fpsyg.2026.1805028

**Published:** 2026-03-24

**Authors:** Tansu Kurtuldu, Samet Sağ, Ebubekir Livmercan, Sabiha Gizem Engin

**Affiliations:** 1 Yozgat Bozok University, Faculty of Sport Sciences, Department of Physical Education and Sports, Yozgat, Türkiye; 2 Yozgat Bozok University, Faculty of Sport Sciences, Department of Sports Management, Yozgat, Türkiye

**Keywords:** dark personality trait, gender equality, gender stereotypes, moral disengagement, perception of female performance athletes

## Abstract

**Purpose:**

The aim of this study is to examine the mediating role of moral disengagement in the effect of dark personality trait on perceptions of female performance athletes, and to test whether this relationship differs according to gender, age, and physical activity level.

**Methods:**

The study was conducted with 726 volunteer participants (362 men, 364 women; age range 18–56; X¯ = 22.59 ± 5.85) collected online using a convenience sampling method. Data were obtained using a Personal Information Form created by the researchers, the Short Dark Triad (SD3-T) Scale, the Moral Disengagement Scale, and the Perception Scale for Female Performance Athletes. Normality and outlier analyses were performed prior to the analysis, and no outliers were detected.

**Results:**

Correlation findings showed a significant positive correlation between dark personality and moral disengagement; and a significant negative correlation between both variables and perceptions of female performance athletes (p < 0.01). Mediation and moderation effects were tested using SPSS PROCESS Macro with a sample of 5,000 bootstraps. In the mediation analysis, it was found that dark personality increases moral disengagement, and moral disengagement decreases perception. When moral disengagement was included in the mediation model, the direct effect of dark personality on perception decreased but remained significant. The indirect effect was confirmed by the bootstrap confidence interval not including zero. In moderation analyses, interactions for gender and physical activity level were not found to be significant, while the interaction for age was found to be significant; simple slope analyses showed that the negative effect of dark personality on perception strengthened with increasing age.

**Conclusion:**

In conclusion, the findings suggest that individuals with dark personality traits utilize moral disengagement strategies to justify negative evaluations of female athletes, a tendency that appears to be more pronounced in older individuals.

## Introduction

Modern sport has witnessed a marked increase in women’s participation; however, performance sport remains far from a domain in which gender equality is fully institutionalized. However, this picture is not a simple phenomenon that can be explained solely in terms of unequal opportunity; rather, within the framework of Social Role Theory, it constitutes a profound reflection of the moral and normative tension between the traditional roles ascribed to women and the competitive nature of sport. Even when the visibility of female athletes increases, this visibility is often framed by sexist stereotypes; while their achievements are coded as “exceptions,” the emphasis placed on the body and femininity—rather than on performance itself—signals an erosion in the ethical values of sport (He et al., 2024; Halıcı et al., 2024; Toffoletti and Thorpe, 2018). Particularly in contact based sports labeled as “masculine,” female athletes face not only competitive pressure but also a legitimacy question grounded in social norms; this makes it necessary to address debates on “moral decline” not merely as an issue of gender inequality, but together with the influence of social acceptances and psychological barriers (Toffoletti and Thorpe, 2018; Glazbrook et al., 2024). Indeed, recent studies indicate that female athletes’ displays of “non-normative” strength and assertiveness lead to their competence being questioned and to the reproduction of psychological pressure (Kuntz and Moorfield, 2024; Toffoletti and Thorpe, 2018).

Such condescending assessments of female performance athletes are related not only to the existence of societal norms, but also to how individuals internalize these norms and transform them into everyday judgments. Certain individual personality traits, in particular, can make comments containing demeaning, accusatory, or demeaning assessments of female athletes more likely. In this context, dark personality traits present a risk profile predisposing individuals to harsher, less empathetic judgments; the cognitive process that allows a person to justify these judgments in their own eyes as “reasonable” and “justified” can be explained by moral disengagement.

Personality traits are one of the important psychological determinants that shape individuals’ attitudes towards social events and groups. In this context, traits referred to as “dark personality traits” in the psychology literature, which can impair interpersonal functioning, are increasingly discussed in research on prejudice and discrimination (Paulhus and Williams, 2002). Dark personality traits offer a holistic framework encompassing Narcissism (superiority complex, expectation of admiration, and entitlement), Machiavellianism (manipulation, strategic thinking, and self-interest), and Psychopathy (impulsivity, insensitivity, and low levels of empathy) (Furnham et al., 2013; Jones and Paulhus, 2014). The common core of this framework points to negative behavioral traits and a more “cold” orientation in social relationships; indeed, meta-analytic findings show that the dark personality trait is consistently associated with lower agreeableness and lower empathy, which in turn can be seen in individuals exhibiting less compassionate and more instrumental patterns in their social judgments (Forsyth et al., 2012). Some studies show that dark personality traits are associated with a desire to maintain social hierarchy and negative attitudes toward egalitarian policies (Dellagiacoma et al., 2024). This hierarchical perspective is also reflected in gender relations; it has been found that dark traits facilitate the objectification of women (Rucobo, 2025) and reinforce the acceptance of hostile sexism and aggressive myths (Galán et al., 2024). Moreover, it has been observed that these individuals can legitimize their prejudices by using sexist humor as a strategic tool to avoid social sanctions (Li et al., 2023). Furthermore, recent research shows that these traits have a positive correlation with general aggression (Polat et al., 2024).

While dark personality traits offer a structural basis explaining individuals’ predisposition to hostile attitudes and behaviors, the fact that these tendencies can be acted upon and sustained without pangs of conscience points to the existence of an intermediary cognitive mechanism. In this context, Albert Bandura’s conceptualization of “moral disengagement” within the framework of Social Cognitive Theory provides a fundamental theoretical framework explaining the process by which an individual temporarily disables their own internal moral standards to legitimize harmful behaviors (Bandura, 2014; Bandura, 2017). Moral disengagement is the process by which individuals make antisocial behavior acceptable to themselves by using cognitive strategies such as moral justification, diffusion of responsibility, distortion of consequences, and dehumanization/blame attribution (Bandura et al., 1996; Moore et al., 2012). Thanks to this mechanism, individuals can maintain their self-esteem even when exhibiting unethical behavior, and can carry out aggressive or discriminatory actions that they would normally avoid, without feeling guilty (Detert et al., 2008). Therefore, moral disengagement should be considered not merely a behavior, but a cognitive catalyst that facilitates the transformation of personality traits into negative judgments against female athletes, thus protecting the individual from self-sanction.

In a world where sport continues to be a battleground for gender equality, understanding the origins of discriminatory attitudes towards female performance athletes requires a multifaceted examination that goes beyond mere sociocultural norms. Although previous studies have generally examined inequality and discrimination against female athletes in the context of sociocultural norms, media representations, and institutional policies (Fink, 2015; Halıcı et al., 2024; Toffoletti and Thorpe, 2018; Bowes and Bairner, 2018; World Athletics, 2019; International Olympic Committee (IOC), 2021), very little is known about how individuals’ dark personality traits shape these negative attitudes and how moral disengagement functions as a mediating mechanism in this process. This study holds a unique position in the literature because it addresses these negative attitudes within the context of dark personality traits and moral disengagement, a cognitive mechanism that translates these traits into action. Another critical contribution of the study is its examination of the impact of exposure to sports culture; including participants’ levels of physical activity (active athletes, individuals engaging in recreational sports, and sedentary individuals) in the model allows for testing possible differences (or similarities) in perspectives between those who are directly involved in sports and those who occupy the role of spectators. Furthermore, examining the moderating effects of gender and age variables will identify which demographic profiles constitute at-risk groups, providing an evidence-based, psychosocial foundation for the inclusion policies and ethical awareness training that sports organizations will develop.

### Research hypotheses

The hypotheses developed in line with the aim and theoretical framework of the research are presented below.

### Main effect and relationship hypotheses

*H1*: The dark personality trait positively and significantly predicts the level of moral disengagement.

*H2*: The level of moral disengagement negatively and significantly predicts perceptions of female performance athletes.

*H3*: The dark personality trait significantly and negatively predicts perceptions of female performance athletes.

### The mediation hypothesis

*H4*: Moral disengagement plays a mediator role in the relationship between dark personality traits and perceptions of female performance athletes.

### The moderation hypotheses

*H5*: Participants’ gender plays a moderating role in the relationship between dark personality traits and perceptions of female performance athletes.

*H6*: Participants’ physical activity levels (active athlete, recreational athlete, and sedentary individual) significantly differentiate the strength of the relationship between dark personality traits and perceptions of female performance athletes.

*H7*: Participants’ age plays a moderating role in the relationship between dark personality traits and perceptions of female performance athletes.

## Method

The study sample consisted of volunteer participants selected using convenience sampling, a type of improbable sampling method. In order to determine the sample size in the study, a power analysis based on multiple linear regression was conducted using the G*Power 3.1 software. The analysis considered the following variables in the model: dark personality trait (independent variable), perception of female performance athletes (dependent variable), moral disengagement (mediating variables), gender and physical activity level (moderating variables), and the interaction terms of these variables. In the power analysis, the effect size ($f^2$) was set at 0.02 (small effect), the significance level ($\alpha$) at 0.05, and the test power ($1-\beta$) at 0.95. The aim of the calculations based on these parameters was to achieve a sufficient sample size to accommodate the predicted complexity and potential interactions of the model. Based on this analysis, the minimum required sample size was calculated to be 652.

A total of 726 individuals participated in the study, exceeding this calculated requirement. The inclusion criteria were: (I) being 18 years of age or older, (II) being able to read and understand Turkish, and (III) providing informed consent. Of the participants, 362 were male (49.9%) and 364 were female (50.1%). Participants’ ages ranged from 18 to 56 years, with a mean age of 22.59 ± 5.85.

### Data collection process and data collection tools

The data collection process for this study was conducted entirely online. Participants completed a form prepared via Google Forms using their computers or smartphones. The purpose of the research was explained at the beginning of the form, followed by an informed consent form and relevant scales. Informed consent was obtained online by asking participants to read the information statement on the first page of the form and then select the option “I approve/I agree”; those who did not provide consent were not directed to the scale pages. Participants were informed that they could withdraw from the study at any time. Because the scale items were set as required responses in Google Forms, no missing data occurred for the questionnaire items. Participants completed the scales in an average of 5–10 min. Online data collection was preferred due to its advantages such as rapid access, reaching a wide audience, ease of data processing, and encouraging voluntary participation (Creswell and Creswell, 2014). The research was conducted in accordance with the ethical principles of the Helsinki Declaration and received ethical approval from the Yozgat Bozok University Social and Human Sciences Ethics Committee with decision number 31/45 dated 17.12.2025.

*Personal information form*: demographic information was collected from participants in the study through a personal information form. Information regarding gender, age, and physical activity level was obtained from the participants.

*Short Dark Triad (SD3-T):* developed by Jonason and Webster (2010), it is used to measure individuals’ dark personality traits. The scale consists of a total of 12 items and three sub-dimensions. The first sub-dimension, “Narcissism,” is exemplified by the item, “I tend to seek prestige or status.” For the second sub-dimension, “Psychopathy,” the example item is, “I tend to not bee too concerned with morality or the morality of my actions. Finally, to measure the third sub-dimension, “Machiavellianism,” the item, “I tend to manipulate others to get my way,” is used. The scale, adapted into Turkish by Özsoy et al. (2017), has a 5-point Likert-type rating scale ranging from ‘1 = Strongly Disagree’ to ‘5 = Strongly Agree’. The total score obtained from the scale, which does not contain any reverse items, ranges from 12 to 60. A higher score indicates that the individual has a greater general tendency towards darkness. However, the Dark Triad literature notes that these three dimensions can be considered not only as related sub-dimensions but also through a total/composite score structure reflecting overall dark personality tendencies (Jonason et al., 2009; Jonason et al., 2010; Tekeş and Bıçaksız, 2021). Therefore, the fact that SD3 can be modeled as both three sub-dimensions and a single overall score is consistent with the literature (Jonason et al., 2009; Jonason et al., 2010). In the study, the Cronbach’s alpha internal consistency coefficient calculated for the scale as a whole was found to be *α* = 0.89. To gain stronger certainty about the scale’s measurement structure in this study and the usability of the total score as a representative of the “general dark characteristic,” a confirmatory factor analysis (CFA) was conducted before proceeding with the analyses. In this context, the fit of the data to a single-factor structure for the scale items was tested, and the model was found to demonstrate acceptable/good fit: *χ*^2^/*df* = 2.40; CFI = 0.97; TLI = 0.96; RMSEA = 0.045; NFI = 0.95; GFI = 0.94; AGFI = 0.91. In light of these findings, the scale was modeled on a total score basis in the analyses to represent the overall level of dark personality traits.

*Moral disengagement scale:* developed by Moore et al. (2012), it is used to measure individuals’ tendencies to justify unethical behavior and not feel guilt about such behavior. The scale consists of a total of 8 items and is of a single dimension. Examples of statements included in the scale are, “There’s nothing wrong with spreading gossip if it protects your loved ones,” and “There’s nothing wrong with taking something without the owner’s permission, as long as you are borrowing it.” The scale, adapted into Turkish by Ekmekçioğlu and Aydoğan (2019), has a 5-point Likert-type rating structure ranging from “1 = Strongly Disagree” to “5 = Strongly Agree”. The total score obtained from the scale, which does not contain any reversing-items, ranges from 8 to 40. A high score on the scale indicates a high level of moral disengagement. In the Turkish adaptation study of the scale, the Cronbach’s Alpha coefficient was reported as 0.91. In this study, however, the Cronbach’s Alpha internal consistency coefficient calculated for the scale as a whole was determined to be 0.88.

*Perception scale for female performance athletes:* developed by Kurşun et al. (2023), it is used to measure gender-based perceptions of performance sports spectators toward female athletes. The scale consists of a total of 23 items and three sub-dimensions. An example of the first sub-dimension, “Cultural Value Judgments,” is the item “I do not find it appropriate for women to participate in sports in front of an spectator.” For the second sub-dimension, “Integration through Social Support,” the example item presented is “I support women participating in sports.” To measure the third sub-dimension, “Body Image,” the item “A muscular body type does not suit women” is used. The scale, which has a 5-point Likert-type rating structure with 1 = Strongly Disagree, 2 = Disagree, 3 = Neutral, 4 = Agree, and 5 = Strongly Agree, has the sub-dimensions of “Cultural Value Judgments” and “Body Image” coded as reverse items, and the total score that can be obtained ranges from 23 to 115. The total score of the scale can also be calculated in the study where the scale was developed (Kurşun et al., 2023). A high score indicates that the individual’s perception of female performance athletes is positive. In this study, the Cronbach’s Alpha internal consistency coefficient calculated for the scale as a whole was determined to be *α* = 0.80. To evaluate the measurement structure of the scale in this study and to test the usability of the total score, confirmatory factor analysis (CFA) was conducted, and the model fit was found to be at an acceptable level: *χ*^2^/*df* = 2.52; CFI = 0.96; TLI = 0.96; RMSEA = 0.041; NFI = 0.95; GFI = 0.92; AGFI = 0.90. In light of these findings, in subsequent analyses the scale was modeled using a total score to represent the overall level of perception.

### Data analysis

Before proceeding with data analysis, the normality assumption of the dataset was evaluated, and outliers were examined using Mahalanobis distance values. The analysis revealed no outliers in the dataset. The analysis identified no outliers in the dataset. In addition, the regression assumptions underlying the mediation analysis were tested. First, the presence of a multicollinearity problem among the independent variables was examined. According to the literature, the VIF (Variance Inflation Factor) value should be below 5 to indicate the absence of this problem (Hair et al., 2019). The analysis yielded VIF values of 2.180 (<3), thus confirming that there was no multicollinearity issue in the model. Second, the Durbin–Watson coefficient was inspected to test the independence of errors (i.e., the absence of autocorrelation). The calculated value of 1.631 fell within the acceptable reference range of 1.5 to 2.5, indicating that this assumption was also met (Jonas et al., 2015).

**Figure 1:**
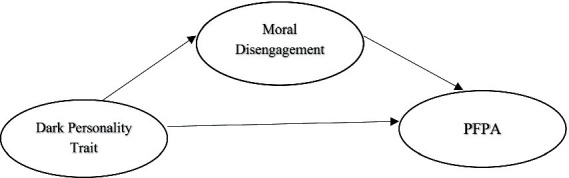
The mediating role of moral disengagement in the perception of dark personality trait for female performance athletes.

In this study, dark personality trait was used as the independent variable, and perception of female performance athletes was used as the dependent variable. Moral disengagement was used as the mediating variable. The structure of the mediation model of the study is presented in Figure 1. Figure 1 shows the mediating role of moral disengagement in the perception of dark personality trait towards female performance athletes.

**Figure 2:**
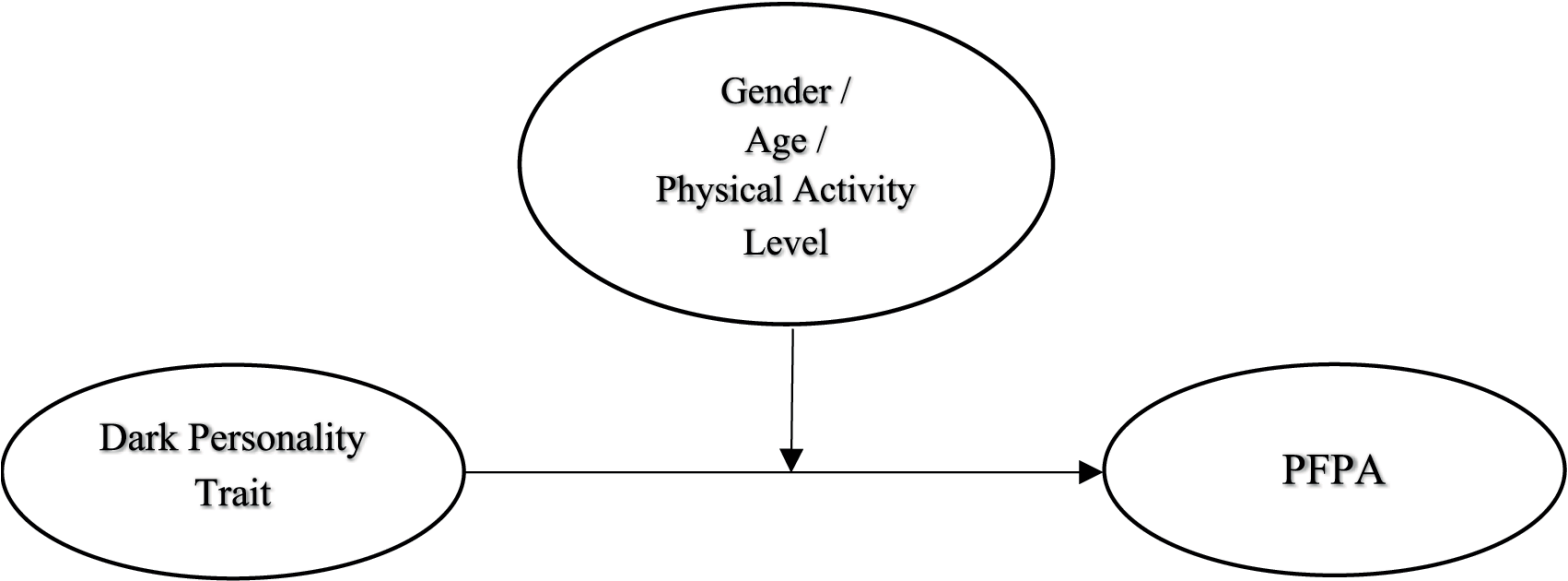
The separate moderating role of gender, age, and physical activity level on the perception of dark personality trait among female performance athletes.

Gender, age, and physical activity level (active athlete, recreational athlete, and sedentary individual) were identified as moderating variables. The study’s model including the moderating effect is shown in Figure 2. Figure 2 shows the separate moderating role of gender, age, and physical activity level on the perception of the dark personality trait among female performance athletes.

Whether the effects in the model were statistically significant was evaluated with a sample of 5,000 Bootstrap. The analyses were performed using the PROCESS Macro (4.2) add-in in IBM SPSS 24 program, with Model 4 chosen for the mediating variable and Model 1 (Hayes, 2012) chosen for the moderating effect. The significance level was accepted as 0.05 in the study.

In analyses conducted under the contemporary approach, even if paths a, b, c, and c′ are not statistically significant, a statistically significant indirect effect based on the bootstrap test is considered sufficient to establish (confirm) mediation (Gürbüz, 2019; Hayes, 2012). The fact that the lower and upper values of the 95% confidence interval for the indirect effect do not contain zero (0) indicates that the mediating effect is significant. In the traditional approach, however, paths c, a and b must be significant in order to speak of a mediating effect (Baron and Kenny, 1986; Gürbüz, 2019). In this study, the contemporary method was preferred.

Process Macro makes it possible to include complex models containing both mediating and moderating variables in the analysis at once and reduce the amount of error (Hayes, 2022). Furthermore, the interaction effects of the moderating variable can be calculated automatically. Therefore, in this study, Process Macro was used to test models containing both mediating and moderating effects.

## Findings

Table 1 presents the descriptive statistics for the variables examined in the study. Analysis results show that participants’ levels of dark personality trait (X¯ =2.50) and moral disengagement (X¯ =2.29) were moderate to below-moderate; however, their perception of female performance athletes (X¯ =3.83) was quite high. When the distribution characteristics of the data were examined, it was determined that the skewness and kurtosis values for all variables were in the range of +1.5 to −1.5. These values are within the normality cutoffs accepted in the literature (Tabachnick and Fidell, 2007), indicating that the data exhibit a normal distribution and are suitable for the application of parametric tests.

**Table 1:** Descriptive statistics of the variables investigated in the study.

Variables	Min	Max	X¯	SD	Skewness	Kurtosis
Dark personality trait	1	5	2.50	0.837	0.043	−0.484
Moral disengagement	1	5	2.29	0.809	0.244	−0.200
Perception of female performance athletes	1.7	5	3.83	0.829	0.048	−1.417

*N*: 726.

The analysis revealed a strong positive correlation between dark personality traits and moral disengagement (*r* = 0.736, *p* < 0.01); that is, as dark traits increase, the level of moral disengagement also increases. Conversely, significant negative correlations were found between both dark personality traits (*r* = −0.508) and moral disengagement (*r* = −0.583) and perceptions of female performance athletes (*p* < 0.01). These findings indicate that as the trait increases, positive perceptions of female athletes decrease, thus fulfilling the necessary relational preconditions for mediation analysis (see Tables 2, 3, 4).

**Table 2:** Correlation coefficients between variables.

Variables	Dark personality trait	Moral disengagement	Perception of female performance athletes
Dark personality trait	1		
Moral disengagement	0.736**	1	
Perception of female performance athletes	−0.508**	−0.583**	1

**p < 0.01.

**Table 3:** Findings regarding the mediating role of moral disengagement in the relationship between dark personality trait and perceptions of female performance athletes.

Paths	B	SE	*t*	%95 CI [lower, upper]
Path a (dark personality trait → moral disengagement)	711	0.024	29.22	[0.663, 0.759]
Path b (moral disengagement → perception of female athletes)	−0.467	0.045	−10.33	[−0.556, −0.379]
Total effect (c) (dark personality trait → perceptions of female elite athletes)	−0.504	0.032	−15.88	[−0.566, −0.441]
Direct effect (c′) (dark personality trait → perceptions of female elite athletes)	−0.171	0.044	−3.91	[−0.257, −0.085]
Indirect effect (mediation) (dark personality trait → moral disengagement → perceptions of female elite athletes)	−0.332	0.038	—	[−0.410, −0.261]

B: Unstandardized regression coefficient; SE, Standard error; CI, Confidence interval. Analyses were conducted using Hayes (2022) PROCESS macro (Model 4). Indirect effect estimates and confidence intervals were computed using 5,000 bootstrap samples.

**Table 4:** The moderating role of gender and physical activity level in the relationship between dark personality trait and perception.

Variables	B	SE	*t*	%95 CI [lower, upper]
Gender as a moderator				
Dart personality trait (X)	−0.351	0.098	−3.58	[−0.543, −0.159]
Gender (W)	0.680	0.159	4.27	[0.367, 0.993]
Dark personality trait × gender	−0.069	0.060	−1.14	[−0.187, 0.050]
The moderating role of physical activity level				
Dark personality trait (X)	−0.408	0.098	−4.18	[−0.600, −0.216]
Physical activity level (W)	0.117	0.129	0.91	[−0.136, 0.370]
Dark personality trait × physical activity level	−0.051	0.049	−1.03	[−0.147, 0.046]

Both analyses were tested separately using Hayes (2022) PROCESS macro (Model 1). B: unstandardized regression coefficient; SE, standard error; CI, confidence interval.

Analysis findings show that the independent variable, dark personality trait, positively and statistically significantly predicts the mediating variable, moral disengagement (B = 0.711, *p* < 0.001). This finding supports hypothesis H1. Conversely, the mediating variable, moral disengagement, was found to negatively and significantly affect the dependent variable, perception of female performance athletes (B = −0.467, *p* < 0.001). Accordingly, H2 is supported. The overall effect of the dark personality trait on perception of female performance athletes was found to be negative and significant (B = −0.504, *p* < 0.001); therefore, H3 is supported. When moral disengagement was included in the model, it was determined that the direct effect of the dark personality trait on perception decreased but retained its significance (B = −0.171, *p* < 0.001). This situation indicates the existence of a partial mediation relationship between the variables. The significance of the mediation effect was tested using the 5,000 bootstrap technique, and the resulting indirect effect coefficient was determined to be −0.332. When the lower and upper limit values of the 95% confidence interval calculated by the bootstrap method [−0.410, −0.261] are examined, it is seen that this interval does not include the value of zero (0). In conclusion, moral disengagement was found to have a significant mediating role in the relationship between dark personality traits and perceptions of female performance athletes, supporting hypothesis H4.

In the moderation analyses performed (Model 1), neither gender (B = −0.069, *p* > 0.05) nor physical activity level (B = −0.051, *p* > 0.05) was found to have a significant moderating role in the relationship between dark personality trait and perceptions of female athletes. Accordingly, hypotheses H5 and H6 were not supported.

According to the moderation analysis results presented in Table 5, the age variable was found to have a statistically significant moderating role in the relationship between the dark personality trait and perceptions of female athletes (B = −0.019, *t* = −3.31, *p* = 0.001). Accordingly, hypothesis H7 is supported. When the results of the conditional effects (simple slope) analysis, which was carried out to determine the nature and direction of this significant interaction, are examined, it is seen that the negative effect of the dark personality trait on the perception of female performance athletes is statistically significant at all age levels (*p* < 0.001), but the intensity of this effect varies with age.

**Table 5:** The moderating role and conditional effects of age in the relationship between dark personality trait and perception.

Variables and levels	B	SE	*t*	%95 CI [lower, upper]
The moderating role of age				
Dark personality trait (X)	−0.062	0.138	−0.45	[−0.333, 0.209]
Age (W)	0.030	0.013	2.24	[0.004, 0.056]
Dark Personality trait × age (interaction)	−0.019	0.006	−3.31	[−0.031, −0.008]
Conditional effects (by age)				
Low age (19)	−0.428	0.039	−10.85	[−0.505, −0.351]
Middle age (21)	−0.466	0.034	−13.84	[−0.533, −0.400]
Relatively older participants (28)	−0.601	0.042	−14.18	[−0.685, −0.518]

Analyses were conducted using Hayes (2022) PROCESS macro (Model 1). *R*^2^ = 0.276, *p* < 0.001. For the conditional effects, age levels were based on the 16th, 50th, and 84th percentile values.

According to the results, among participants in the low-age group (19 years), the negative effect of dark personality trait on perceptions of female performance athletes was B = −0.428; this effect increased to B = −0.466 in the middle-age group (21 years) and to B = −0.601 in the relatively older participants (28 years). This increase in coefficients shows that as the age of the participants increases, the diminishing effect of dark personality traits on the perception of female athletes becomes stronger and more pronounced. In other words, as age increases, the negative perceptions stemming from individuals’ dark personality traits are reflected more harshly.

## Discussion and conclusion

The research findings indicate that moral disengagement plays a significant and partial mediating role in the relationship between dark personality traits and perceptions of female performance athletes. The primary reason why moral disengagement, as a psychological mechanism, may assume a mediating role in this relationship may be that individuals with Dark Triad traits tend to instrumentalize others and exhibit empathic deficits. When openly displaying hostile or prejudiced attitudes, an individual may activate moral disengagement strategies to protect their self-esteem and avoid social condemnation. Thus, moral disengagement can function as a cognitive bridge between the destructive impulses of dark personality and discriminatory perceptions toward women, legitimizing within the individual’s inner world attitudes that would normally be considered unacceptable. This finding suggests that as the level of dark personality traits increases, individuals not only directly reflect their negative and demeaning evaluations of female athletes but also cognitively justify and legitimize these negative perceptions through moral disengagement mechanisms. The characteristics of the dark personality trait, such as low empathy and lack of emotion, can make individuals more susceptible to unethical tendencies. This is strongly emphasized both in the definition of the concept and in discussions regarding the measurement of its sub-dimensions (Paulhus and Williams, 2002; Jones and Paulhus, 2014). Interpreted within Bandura’s Social Cognitive approach, the dark personality trait’s positive prediction of moral disengagement is consistent with individuals’ tendency to temporarily disable self-control and self-sanction processes, thus “justifying” their negative judgments (Bandura, 2017). Evaluations that belittle the performance of female athletes, overshadow their achievements, or reduce them to physical appearances can be re-justified through moral disengagemen strategies that help individuals perceive their own attitudes as more “acceptable.” The literature shows that these justifications can be grounded particularly in cognitive mechanisms such as minimizing the harm, diffusion/displacement of responsibility, and blaming the target/victim; thus, they can reduce an individual’s moral discomfort and facilitate the continuation of negative judgments (Corrion et al., 2009; Boardley and Kavussanu, 2011; Kavussanu and Ring, 2017). Evidence suggesting that moral disengagement in the context of sport is associated with antisocial tendencies and unsportsmanlike patterns, and that moral disengagement functions as a cognitive process that can “contextually” loosen behaviors and evaluations in sport, supports the consistency of the current mediating outcome with the literature (Boardley et al., 2020; Gentile et al., 2022; Hodge and Gucciardi, 2015; Stanger et al., 2024). When the quantitative dimension of the mediation analysis is examined, the difference between the total effect of the dark personality trait on the perception of female performance athletes (B = −0.504) and its direct effect (B = −0.171) when moral disengagement is controlled is noteworthy. This comparison reveals that approximately 66% of the effect occurs through the moral disengagement mechanism. The fact that the bootstrap confidence interval for the indirect effect (B = −0.332) does not include zero [−0.410, −0.261] supports the statistically reliable nature of this mediating effect. On the other hand, the fact that the direct effect is still significant in the model (i.e., partial mediation) indicates that negative attitudes towards female athletes are not formed solely through moral disengagement. The broader emotional and social impacts of the dark personality trait, as well as gender-based stereotypes present in sports media and society, can also fuel this negative perception. As an alternative explanation, it should not be overlooked that other ideological and psychological mechanisms—such as social dominance orientation or hostile sexism—may also play a role in this partial mediation relationship (Navas et al., 2020). Individuals with dark personality traits may also be shaping their negative perceptions of female athletes through social dominance tendencies that view hierarchy and inequality as legitimate. In this context, research that reveals how female athletes are portrayed in the media and how discussions about gender equality are intertwined with media narratives becomes important (McGannon et al., 2024). Furthermore, recent meta-analysis studies examining the psychological and social factors influencing unethical behavior tendencies in sports (Ntoumanis et al., 2024) show that negative perception is based not on a single cause, but on many different factors. In addition, recent research reveals that individuals with dark personality traits can normalize prejudice through sexist jokes or domineering attitudes (Li et al., 2023; Dellagiacoma et al., 2024). When all these findings are considered together, the partial mediation model obtained points to an important conclusion: discriminatory perceptions towards female performance athletes can persist, fueled by both personality-based biases and the moral desensitization processes triggered by these biases. This indicates that the research makes a significant contribution to the literature.

This study investigated whether gender and physical activity level (active athlete, recreational athlete, and sedentary individual) played a moderating role in the relationship between dark personality traits and perceptions of female performance athletes. Moderation analyses using Hayes (2022) PROCESS Macro Model 1 showed that the dark personality trait significantly and negatively predicted the perception of female athletes; however, neither gender (B = −0.069, *p* > 0.05) nor physical activity level (B = −0.051, *p* > 0.05) was found to have a significant moderating effect on this relationship. Despite this finding, the possibility of a moderating effect, particularly in the gender variable, was theoretically considered reasonable when establishing the research design; because gender roles and the “gendered” nature of sport suggest that attitudes towards female performance athletes may be shaped through different normative expectations and stereotypes in women and men (Sunderji et al., 2024). Indeed, when the sample distribution is examined, it is seen that the number of female participants is 364 and the number of male participants is 362, and the sizes between the groups are quite similar. This relatively balanced distribution was considered a condition that could increase the likelihood of capturing a statistically significant interaction effect; a moderating pattern was expected, particularly in favor of female participants (which could create a more protective/affirmative tendency in women’s perceptions of female performance athletes). Similarly, regarding physical activity level, there was a justifiable expectation that exposure to and participation in sports could improve the perception of female performance athletes. Accordingly, it was predicted that active athletes might have more positive perceptions of female performance athletes; individuals engaging in recreational sports might have more moderate/level-of-positive perceptions; and sedentary individuals might exhibit relatively lower levels of positive perception. However, the insignificant results of the moderation analyses indicated that the strength of the dark personality-perception relationship did not diverge as expected among activity level groups. This suggests that participating in sport or being exposed to sport alone may not automatically weaken negative evaluation tendencies associated with “dark personality.” When considered from an analytical perspective, this non-significance suggests that dark personality traits—particularly manipulation and a lack of empathy—are embedded in an individual’s cognitive schemas so deeply and rigidly that environmental and demographic factors, such as gender identification or socialization within sport settings, may be insufficient to loosen this rigid structure. Dark personality traits are a personality pattern consisting of Machiavellianism, Narcissism, and Psychopathy, characterized by coldness, self-interest, and manipulative behavior in interpersonal relationships. When these traits are considered together, it is theoretically expected that the primary effect on perception will be negative (Paulhus and Williams, 2002). Studies in the literature also show that dark personality traits are consistently associated with low agreeableness and negative social outcomes (Muris et al., 2017). Furthermore, meta-analytic research supports these findings by showing that this personality structure is linked to norm violations and antisocial behavior (Forsyth et al., 2012). On the other hand, the statistically insignificant results of the interaction terms (confidence intervals including zero) indicate that the negative impact of dark personality traits on perceptions of female performance athletes operates similarly in both female and male participants, as well as in groups with different activity levels. Although some studies have shown that attitudes towards female athletes may differ according to gender (Sunderji et al., 2024), this research found that the dark personality trait exhibited a consistent pattern in its effect on perceptions of female performance athletes, independent of the aforementioned demographic variables. This result suggests that the dark personality trait may function as a context-independent, universally applicable mechanism for shaping negative evaluations of female athletes. It is also noteworthy that physical activity level did not show a moderating effect. Active athletes, recreational participants, and sedentary individuals all exhibited similar levels of negative perception regarding the dark personality trait. This situation reveals that exercise alone cannot mitigate the negative effects of dark personality traits, and that intervention programs in this area should directly target personality-based thought processes.

The study found that age was a significant moderating variable in the relationship between dark personality trait and perception of female performance athletes. The significant result of the interaction term in the moderation analysis conducted with Hayes (2022) PROCESS Macro Model 1 (Dark Personality Trait × Age: B = −0.019; *p* < 0.05) indicates that the negative effect of dark personality trait on perception varies according to age level. In other words, as the dark personality trait increases, the perception of female athletes becomes more negative; however, the severity of this negativity varies depending on age. Within the framework of the lifespan development approach, it is emphasized that with age, individuals’ social cognitive processes, especially their responses to negative/maladaptive traits, can become more selective, consistent, and distinct patterns; therefore, the effect of the same personality trait on social perception can differ according to age groups (Baltes, 1987; Carstensen et al., 1999; Charles and Carstensen, 2010). This suggests that while individuals with relatively higher scores may exhibit fewer dark personality traits, their presence can make the impact of these traits on social perception more pronounced. Indeed, Hartung et al. (2022), in their comprehensive study examining age and gender differences in socially negative personality traits, revealed that these traits follow a lifelong developmental course and that age significantly shapes how these traits are expressed. The strengthening of the moderating effect of age in the current research can also be explained from a psychosocial maturity perspective; Steinberg and Cauffman (1996) showed that judgment maturity is related not only to cognitive factors but also to psychosocial dimensions such as responsibility, moderation, and perspective-taking. In this context, the fact that older individuals give more consistent and distinct responses in their social evaluations can be considered a reflection of their enhanced psychosocial maturity; in other words, older individuals are able to perceive dark personality traits more clearly and develop more definite attitudes towards these traits. Similarly, in their study examining the effect of age on social evaluations, Hess and Auman (2001) found that older individuals develop more consistent and definitive judgments when forming impressions based on personality traits, a tendency particularly evident in the evaluation of negative traits. Considering these findings together, it can be said that as individuals age, they develop more ingrained schemas regarding both dark personality traits and gender norms, and these schemas are more strongly at play in evaluations of female athletes.

This research revealed that dark personality traits negatively influence perceptions of female performance athletes, with moral disengagement acting as a partial mediator and age as a significant moderator in this relationship. The findings indicate that individuals with dark personality traits can cognitively justify their negative evaluations of female athletes through moral disengagement mechanisms; and that this negative perception becomes more pronounced and consistent with age. On the other hand, the lack of a moderating effect of gender and physical activity level on this relationship suggests that the dark personality trait’s influence on negative perceptions of female athletes operates as a generalized mechanism independent of demographic and contextual factors. From a theoretical perspective, this study offers an analytical framework for the cognitive underpinnings of gender-based biases in sport by integrating Bandura’s moral disengagement theory with the dark personality literature.

## Recommendations

The results of this study provide important applied implications for intervention programs aimed at preventing negative perceptions of and discrimination against female performance athletes. Accordingly, it is recommended that, given that perceptions may become more negative as individuals’ dark personality tendencies increase, sports settings and universities should implement structured educational content grounded directly in respect and sports ethics rather than relying solely on superficial equality messages. Moreover, considering the mediating role of moral disengagement in this relationship, it is necessary to develop awareness-raising and reflective psychological content that directly targets the cognitive distortions individuals use to legitimize their negative judgments (e.g., harm-minimizing claims such as “it’s being exaggerated,” victim-blaming justifications such as “she deserved it,” or rationalizations such as “it does not do any harm”). In addition, since the negative effect of dark personality tendencies on perceptions strengthens with age, awareness initiatives should not be limited to younger athletes but should include tailored messages and examples for older age groups. Finally, because gender and physical activity level do not appear to create a meaningful difference in this mechanism, preventive efforts should be designed to reach the general public in an inclusive manner (women and men, those who exercise and those who do not), rather than targeting only a specific demographic group.

## Limitations

While the findings of this study offer valuable insights, some limitations should be considered: Because the research was conducted using a cross-sectional design, the relationships between variables cannot be interpreted causally; furthermore, the use of online and convenience sampling may limit the generalizability of the sample. The measures used are self-reported; this may be susceptible to biases such as social desirability, common methodological bias, and participants misrepresenting themselves. Although the study modeled the SD3-T through a total score of “general dark personality,” examining the relationships between the Narcissism, Machiavellianism, and Psychopathy sub-dimensions and perceptions of female athletes separately could provide more detailed interpretations. Furthermore, since the research is based solely on quantitative data, further studies using qualitative methods such as in-depth interviews/focus groups and with different sample groups (e.g., different age ranges, sports branches, sports audience-athlete distinction) are recommended to better understand the rationale behind perceptions of female performance athletes and how moral disengagement mechanisms operate.

## Data Availability

Publicly available datasets were analyzed in this study. This data can be found at: https://osf.io/e7a5m/overview?view_only=5431769e473c403e973d060f32f1869d.
